# Providing cell phone numbers and email addresses to Patients: the physician's perspective

**DOI:** 10.1186/1756-0500-4-76

**Published:** 2011-03-23

**Authors:** Roni Peleg, Angelika Avdalimov, Tamar Freud

**Affiliations:** 1 Department of Family Medicine and Siaal Research Center for Family Practice and Primary Care, Faculty of Health Sciences, Ben Gurion University of the Negev, Beer Sheva, Israel

## Abstract

**Background:**

The provision of cell phone numbers and email addresses enhances the accessibility of medical consultations, but can add to the burden of physicians' routine clinical practice and affect their free time. The objective was to assess the attitudes of physicians to providing their telephone number or email address to patients.

**Methods:**

Primary care physicians in the southern region of Israel completed a structured questionnaire that related to the study objective.

**Results:**

The study population included 120 primary care physicians with a mean age of 41.2 ± 8.5, 88 of them women (73.3%). Physicians preferred to provide their cell phone number rather than their email address (P = 0.0007). They preferred to answer their cell phones only during the daytime and at predetermined times, but would answer email most hours of the day, including weekends and holidays (P = 0.001). More physicians (79.7%) would have preferred allotted time for email communication than allotted time for cell phone communication (50%). However, they felt that email communication was more likely to lead to miscommunication than telephone calls (P = 0.0001). There were no differences between male and female physicians on the provision of cell phone numbers or email addresses to patients. Older physicians were more prepared to provide cell phone numbers that younger ones (P = 0.039).

**Conclusions:**

The attitude of participating physicians was to provide their cell phone number or email address to some of their patients, but most of them preferred to give out their cell phone number.

## Background

The compulsory national health insurance system, implemented in Israel in 1995, provides health care services to the entire population through nonprofit health maintenance organizations (health funds). The ongoing competition between the various health maintenance organizations (HMOs) motivates them to improve healthcare services and enhance patient satisfaction by ongoing innovation and increased efficiency. The challenge is to do this while meeting budget constraints on the one hand and continuing out to look out for patients' interests on the other. These circumstances have placed an emphasis on the issue of providing patients with their physician's personal cell phone number or email address.

In an attempt to improve quality of care in the face of increasing workloads, clinicians use innovative methods to deliver care, including expanded use of telephone consultations [1]. A number of studies have demonstrated that telephone consultations are shorter than face to face consultations [2,3], and may be time effictive in managing chronic illness [4]. Used appropriately, telephone consultations can enhance access to health care, aid continuity of care, and save time and travel for patients [5].

Patients who visit primary care clinics also contact their physicians frequently by phone [6]. One study showed that 83.1% of telephone consults did not require a clinic visit as the problem was solved over the phone. It was possible to follow-up on 58.2% of the cases through the cell phone alone [7]. Most family physicians define telephone consultations as a reliable service [8].

Electronic communication promises to revolutionize the delivery of health care [9]. In a review of patient-physician communication, patients were reported to be satisfied with communication with their physicians through email, which they found to be convenient and useful. No adverse effects were reported by physicians [10]. In another review of the role of email in patient-provider communication the authors stated that email is transforming the relationship between patient and providers. The rigorous exploration of the pros and cons of electronic interaction in health care setting will help mark email communication as a more powerful, mutually beneficial tool for the provision of health care [11].

A study that evaluated the experience of physicians who use email for communication with patients found that the most important reasons for using email with patients, among those physicians who were satisfied with its use, were "time saving" (33%) and "helps deliver better care" (28%). compared with "patients requested" (80%) among those who were not satisfied with its use [12].

In another assessment of the use of email with patients physicians reported high levels of satisfaction with this form of communication. although they were concerned about the confidentiality of email communication, few discussed this issue with their patients [13].

To obtain optimal benefit from this service it is important that physicians understand its advantages and limitations. Although providing the telephone number [14] or email address [12] to patients is easy and makes medical consultations more accessible, it also can make the physician's routine workload heavier and interfere with their free time [15].

Informal consultations between patients and physicians are commonplace as are informal consultations among physicians relating to their patients [16]. Email consultations are also common as a means of communication for the same purpose [17].

The primary objective of the present study was to assess the attitudes of physicians to providing their telephone number or email address to their patients. Secondary objectives were: a) to evaluate the advantages and disadvantages of using email and cell phone services; b) to find an optimal framework for these services without negatively affecting either the quality of service or the lifestyle of the physician; and c) to assess differences in attitudes among physicians in terms of age, gender, ethnic background, and work seniority.

## Methods

The study instrument was a self-administered questionnaire that was completed by primary care physicians (seniors and residents) working for the Clalit Health Services in the Negev region. The questionnaires were distributed either at a continuing medical education session or at a study session for residents. It included demographic data on the physician and questions on attitudes regarding the provision of telephone numbers or email address to their patients. The questionnaire (Appendix 1) was formulated after a comprehensive review of the literature. A pilot study was conducted and the questionnaire was revised in light of its results, but no formal validation was done.

Statistical analyses were performed using SPSS software, version 15 (SPSS, Chicago, IL, USA). Chi-square tests were used to analyze statistically significant differences of categorical variables. Two-tailed p values less than 0.05 were considered statistically significant, with a power of 0.8.

## Results

### Socio-demographic characteristics of the study population

The study population was comprised of 120 primary care physicians with a mean age of 41.2 ± 8.5, 88 of them women (73.3%). Seventy (58.8%) were experts in family medicine, and 94 (78.3%) worked in urban clinics. The socio-demographic characteristics of the study participants are shown in Table 1.

**Table 1:** Socio-demographic characteristics of the study population (N = 120).

Variable	Result
**Age in years (N = 118)**	
Mean ± SD	41.2 ± 8.5
Range	26-57

**Gender**	
Male	32 (26.7%)
Female	88 (73.3%)

**Family status**	
Single	10 (8.3%)
Married	102 (85.0%)
Divorced	8 (6.7%)

**Country of medical studies**	
Israel	17 (43.6)
Former Soviet Union	11 (28.2)
Other	11 (28.2)
Total	39

**Primary place of work**	
Urban clinic	94 (78.3%)
Rural clinic/kibbutz	26 (21.7%)0 0

**Seniority as primary care physician (yrs)**	
Mean ± SD (yrs)	12.4 ± 7.7
Range	0-35

**Present work status**	
Clinic director	11 (9.2%)
Specialist in family medicine	59 (49.6%)
Resident in family medicine	48 (40.3%)
General practitioner	1 (0.8%)

### Attitudes towards providing telephone numbers to patients (Table 2)

**Table 2:** Attitudes relating to provision of cell phone numbers to patients.

Question	N (%)
**Do you have a cell phone?**	
Yes	120 (100)

**Do you give your cell phone number to patients?**	
To none	69 (57.5)
To a small portion of them	38 (31.7)
To most of them	11 (9.2)
To all those who are interested	0
Only to those I want to follow closely	2 (1.7)

**What is your attitude towards providing your personal cell phone number to patients?**	
Not prepared to provide it	42 (35.7)
Prepared, but only in special cases	64 (53.3)
Prepared, but only to some of my patients	12 (10.0)
Prepared to give it to all patients	2 (1.7)

**At what times would you agree to get call from your patients?**	
Only at pre-determined times	41 (52.6%)
At most times (other than weekends and holidays	21 (26.9%)
At all times including weekends and holidays	7 (9.0%)
Only during work hours and only if I'm free to answer	9 (11.5%)

**What might make you more amenable to providing your cell phone number to patients? (More than one answer possible)**	
Cell phone provided by my employer	53 (44.2%)
Extra pay for the service	84 (70.0%)
Allotment of time during work to answer calls	60 (50.0%)
Possibility of turning the phone off after work/nothing will make me amenable	2 (1.7%)

All study physicians had cell phones, but 69 (57.5%) did not give their number to patients and 38 of them (37.1%) gave them only to a small percentage of their patients.

Forty of the physicians (51.3%) who were prepared to give their cell phone number to patients thought that providing the number could reduce the number of clinic visits and 37 (47.4%) believed that it could give patients a feeling of security even if they don't use it to call the physician. Forty-one physicians (52.6%) were willing to answer phone calls from patients at pre-determined times while 21 (26.9%) were willing to receive calls during most of the hours of the day, but not on weekends or holidays.

Among the 78 physicians who were prepared to provide their cell phone numbers, 40 (51.3%) said that this could reduce the number of clinic appointments and 37 (47.4%) said that providing cell phone numbers could enhance patients' feelings of personal security even if they don't actually use it.

Among the reasons given for not providing cell phone numbers to patients, 92 (77.3%) said that in cases of medical emergency patients should go to the hospital and 77 (64.7%) said that it would impinge upon their privacy beyond formal work hours.

### Attitudes towards providing email addresses to patients (Table 3)

**Table 3:** Attitudes relating to provision of email addresses to patients.

Question	N (%)
**Do you give your email address to patients?**	
To none	62 (51.7)
To a small portion of them	55 (45.8)
To all those who are interested	3 (2.5)

**What is your attitude towards providing your work email address to patients?**	
Not prepared to provide it	52 (43.3)
Prepared, but only in special cases	45 (37.5)
Prepared, but only to some of my patients	15 (12.5)
Prepared to give it to all patients	4 (3.3)
Prepared even though I think it is unnecessary/I don't use email/it's dangerous and could lead to hacking	4 (3.3)

**At what times would you agree to answer emails from your patients?**	
Only at pre-determined times	13 (19.1%)
At most times (other than weekends and holidays	49 (72.1%)
At all times including weekends and holidays	2 (2.9%)
When I can	4 (5.9%)

**What might make you more amenable to providing your cell phone number to patients? (More than one answer possible)**	
Extra pay for the service	53 (44.2%)
Allotment of time during work to answer emails	84 (70.0%)
Patients are told that there will be a low availability rate	1 (0.8%)

Fifty-five physicians (45.8%) declared that they give their email address to a small percentage of their patients, while 3 (2.5%) refused to give out their email address to anyone who requested it. Among the 68 physicians who were prepared to provide their email address to patients, 54 (79.4%) believed that providing their email address would give a sense of personal security to patients, even if they didn't actually use it. Thirty-nine physicians (57.4%) believed that provision of their email address to patients could reduce the number of unnecessary emergency room visits.

Among the 68 physicians who were prepared to provide their email addresses, 54 (79.4%) said that providing cell phone numbers could enhance patients' feelings of personal security even if they don't actually use it, 25 (36.8%) said that this could reduce the number of clinic appointments, and 23 (33.8%) said that providing the email address was an effective method of communication that could solve patients' problems.

Among the reasons given for not providing email addresses to patients, 77 (65.3%) said that this would prevent physical examination of patients and lead to medical errors, 69 (58.5%) said that in cases of medical emergency patients should go to the hospital, and 67 (56.8%) said that email communication could lead to miscommunication and litigation for medical negligence.

### Comparison of attitudes towards providing cell phone numbers or email addresses to patients (Table 4)

**Table 4:** Comparison of attitudes relating to provision of cell phone numbers and email addresses to patients.

Question	Cell phone N (%)	Email N (%)	P
**All participants**			

**Do you give your cell phone number or email address to patients?**			
To none	69 (58.5)	62 (53.0)	
To a small portion of them	38 (32.2)	55 (47.0)	0.0007
To all those who are interested	11 (9.3)	0 (0)	

**What is your attitude towards providing your cell phone number or work email address to patients?**			
Not prepared to provide it	42 (35.0)	52 (43.3)	0.18
Prepared, but only in special cases	78 (65.0)	68 (56.7)	

**Only physicians prepared to provide their cell phone number (N = 78) or email address (N = 68) to patients**			

**Why do you think it is worthwhile to give your cell phone number or email address to patients? (More than one answer is possible)**			
Can improve the quality of the patient-physician relationship	8 (10.3)	9 (13.2)	0.58
Can give patients a sense of security even if they don't use it	37 (47.4)	54 (79.4)	0.0001
Is an effective tool for solving patients' problems	22 (28.2)	23 (3.8)	0.46
Can reduce the number of clinic visits	40 (51.3)	25 (36.8)	0.08
Can reduce the number of unnecessary emergency room visits (cut costs)	37 (47.4)	39 (57.4)	0.23
A convenient, non-committal means of communication/cannot cause harm	0 (0)	2 (2.9)	0.21
Only in medical emergencies such as changing Coumadin dose with INR result	8 (10.3)	0 (0)	0.00

**At what times would you agree to get call from your patients?**			
Only at pre-determined times	41 (52.6)	13 (19.1)	0.0001
At most times (other than weekends and holidays	21 (26.9)	49 (72.1)	0.0001
At all times including weekends and holidays	7 (9.0)	2 (2.9)	0.12
Only during work hours and only if I'm free to answer	9 (11.5)	4 (5.9)	0.23

**Why do you think it is not worthwhile to give your cell phone number or email address to patients? (More than one answer is possible)**			
Impinges upon my privacy after work hours	77 (64.7)	56 (47.5)	0.007
There are medical services available after clinic hours	52 (43.7)	48 (40.7)	0.64
In a medical emergency it's possible to call an ambulance or go to the emergency room	92 (77.3)	69 (58.5)	0.0002
The inability to conduct a physical examination could cause an error	75 (63.0)	77 (65.3)	0.72
Miscommunication leading to litigation for medical negligence can occur	35 (29.4)	67 (56.8)	0.0001
Pressure/clinic hours are enough for talks	2(1.7)	0(0)	0.25
I don't check email often/there is a danger of hacking	0(0)	7(5.9)	0.007

**What might make you more amenable to providing your cell phone number or email address to patients? (More than one answer possible)**			
Cell phone provided by my employer	53(44.2)		---
Extra pay for the service	84(70.0)	36(30.5)	0.001
Allotment of time during work to answer emails	60(50.0)	94(79.7)	0.001
Possibility of turning the phone off after work, or nothing will make me amenable	2(1.7)	0(0)	0.25
Patients are told that there will be a low availability rate	0(0)	1(0.8)	0.49

Physicians preferred to provide their cell phone number rather than their email address (P = 0.0007). Among the participating physicians 79.4% thought that giving out email addresses to patients could give them a greater sense of security compared to cell phone numbers, even if they didn't actually use them (47.4%, P = 0.001). The physicians preferred to answer their cell phones only during the daytime and at predetermined times, but would answer email most hours of the day, including weekends and holidays (P = 0.001). They thought that providing email addresses would affect their privacy more negatively than cell phone numbers (P = 0.07). They also felt that email communication was more likely to lead to miscommunication than telephone calls (P = 0.0001).

### Comparison of socio-demographic data and attitudes towards providing cell phone numbers or email addresses to patients

There were no differences between male and female physicians on the provision of cell phone numbers (p = 0.233) or email addresses to patients (p = 0.162). Older physicians were more prepared to provide cell phone numbers that younger ones (42.4 ± 7.9 years vs. 39.0 ± 9.3 years, P = 0.039), but there was no significant difference by age in relation to email addresses (41.9 ± 7.7 years vs. 40.1 ± 9.4, p = 0.244).

There was a trend for physicians who completed medical school in countries outside of Israel to be more prepared to provide cell phone numbers and email addresses than those who studied in Israel. In all, 85.9% of the physicians who were prepared to provide cell phone numbers vs. 71.4% of the physicians who were not (p = 0.054), and 86.8% of the physicians who were prepared to provide email addresses vs. 73.1% of the physicians who were not completed medical school in countries outside of Israel (p = 0.06). There was no statistically significant difference in the attitudes of specialists and non-specialists in relation to provision of either cell phone numbers or email addresses.

## Discussion

Today, patients can turn to their physicians for medical advice by cell phone or email. In an effort to improve the quality of healthcare services clinicians use innovative methods of care delivery including consultation by means of cell phones and email [5]. In several countries, requests for medical treatment outside of regular work hours are routinely triaged by telephone [5,18]. In the UK, a national survey showed that patients increasingly want to be able to communicate with health care workers by email [19].

In this study we tried to characterize physicians' attitudes to the provision of cell phone numbers or email addresses to their patients. Most of the participating physicians preferred to give out their cell phone number rather than their email address. There were two major findings in a previous study conducted in a medical center (not a community setting) in Pennsylvania on the provision of cell phone numbers: a) patients believe that this acts shows a greater concern for them on the part of the physician, and b) patients make good, appropriate use of this option when they need it [20]. This form of communication is perceived as a means to improve the quality of patient care and as a way to receive feedback from patients in a way that is beneficial to physicians [21].

In general, patient satisfaction is high when patients have the opportunity to contact the treating physician by telephone [5,8]. In our study physicians stated that they prefer to answer phone calls during daily hours or at predetermined times. In contrast, communication by email provides greater flexibility. The provision of cell phone numbers and email addresses can give patients a greater sense of personal security even if they do not actively use them. If used, the number of unjustified emergency room visits and unnecessary primary care clinic visits can be reduced together with the physician's work burden. Indeed, in another study cell phone calls to physicians significantly reduced the number of emergency room visits [22]. Communication with physicians by email was only studied at a later stage. although a report on communication by email among physicians to discuss patient cases appeared over a decade ago [17].

Today, millions of human beings around the world have access to the internet and use it increasingly. Medical consultation by way of email can play an important role in healthcare, but we still lack sufficient evidence through controlled trials of its potential benefits and how it can be used in routine clinical practice [9].

Together with the advantages cited above there are also disadvantages such as the intrusion into physicians' privacy during off-work hours, interference during other patients' clinic visits, and the danger of miscommunication and medical error [23]. Allocating resources, such as work time or reimbursement for these services could improve physician compliance [24].

This study has several limitations. A sample size could not be pre-determined, so we decided on 120 participating physicians. The study may have been underpowered for some of the statistical analyses. The study population was a convenience sample. Most of the participating physicians answered the questionnaire at continuing education meeting for family physicians. It is conceivable that their attitudes are somewhat different from physicians who do not come to these study days, which could lead to biased results.

Since this study assessed attitudes it does not necessarily reflect actual clinical practice. The best way to evaluate actual clinical practice would be at the clinic during routine clinical work, but this approach would necessitate much greater resources, which were not available to the investigators at the time the study was conducted. The study was conducted in one geographical region in Israel. Local results cannot necessarily be generalized to other areas.

## Conclusions

The attitude of participating physicians was to provide their cell phone number or email address to some of their patients, but most of them preferred to give out their cell phone number. With new technology such as cell phones and email becoming more available and widely used, it is important to understand the significance of integrating these means into clinical practice as well as how that integration should be accomplished. We believe that the results of the present study will make a contribution to understanding these new means of communication and to planning their integration into clinical practice for the improvement of the quality of patient care.

## Competing interests

The authors declare that they have no competing interests.

## Authors' contributions

All authors contributed equally to the study from its initial planning states and through to the writing of this paper.

## Appendix 1

### The physicians' attitude to providing patients with cell phone numbers or email addresses

Dear doctor,

We are conducting a survey on the attitudes of physicians to providing personal cell phone numbers and email addresses to patients so they can use them to consult with their family physicians. The study is being conducted in the framework of the course in **Research Practice **of the Department of Family Medicine. All the data will be safeguarded and kept secret and will only serve for research purposes. We thank you for your cooperation.

1. **Do you have a cell phone?**   1. Yes   2. No

2. **What is your attitude towards providing your personal cell phone number to patients?**

1. Not prepared to provide it

2. Prepared, but only in special cases

3. Prepared, but only to some of my patients

4. Prepared to give it to all patients

3. **Why do you think it is worthwhile to give your cell phone number to patients? (More than one answer is possible)**

1. Can improve the quality of the patient-physician relationship

2. Can give patients a sense of security even if they don't use it

3. Is an effective tool for solving patients' problems

4. Can reduce the number of clinic visits

5. Can reduce the number of unnecessary emergency room visits (cut costs)

4. **Why do you think it is not worthwhile to give your cell phone number to patients? (More than one answer is possible)**

1. Impinges upon my privacy after work hours

2. There are medical services available after clinic hours

3. In a medical emergency it's possible to call an ambulance or go to the emergency room

4. The inability to conduct a physical examination could cause an error

5. Miscommunication leading to litigation for medical negligence can occur

6. Other ______________________________

5. **At what times would you agree to get call from your patients?**

1. I am not prepared to give my cell phone number to patients

2. Only at pre-determined times

3. At most times (other than weekends and holidays)

4. At all times including weekends and holidays

5. Other ________________________

6. **What might make you more amenable to providing your cell phone number to patients? (More than one answers possible)**

1. Cell phone provided by my employer

2. Extra pay for the service

3. Allotment of time during work to answer emails

4. Other _______________________________

7. **Do you give your cell phone number to patients?**

1. To none

2. To a small portion of them

3. To most of them

4. To all those who are interested

8. **Do you use email at work**?

1. Yes

2. No

9. **What is your attitude towards providing your work email address to patients?**

1. Not prepared to provide it

2. Prepared, but only in special cases

3. Prepared, but only to some of my patients

4. Prepared to give it to all patients

5. Other __________________

10. **Why do you think it is worthwhile to give your email address to patients? (More than one answer is possible)**

1. Can improve the quality of the patient-physician relationship

2. Can give patients a sense of security even if they don't use it

3. Is an effective tool for solving patients' problems

4. Can reduce the number of clinic visits

5. Can reduce the number of unnecessary emergency room visits (cut costs)

6. Other _________________

11. **Why do you think it is not worthwhile to give your email address to patients? (More than one answer is possible)**

1. Impinges upon my privacy after work hours

2. I do not have either the time of the resources to relate to patient email messages during work hours

3. There are medical services available after clinic hours

4. In a medical emergency it's possible to call an ambulance or go to the emergency room

5. The inability to conduct a physical examination could cause an error

6. Miscommunication leading to litigation for medical negligence can occur

7. Other ____________________

12. **At what times would you agree to answer emails from your patients?**

1. I am not prepared under any circumstances to give my email address to patients

2. Only at pre-determined times

3. At most times (other than weekends and holidays

4. At all times including weekends and holidays

5. I am prepared to answer emails only if time is allotted for this purpose

6. Other ________________

13. **What might make you more amenable to providing your cell phone number or email address to patients? (More than one answer possible)**

1. Extra pay for the service

2. Allotment of time during work to answer emails

3. Other___________

14. **Do you give your email address to patients?**

1. to none

2. To a small portion of them

3. To most of my patients

4. To all those who are interested

15. **Do you have another email address (personal)?**   1. Yes   2. No

16. **Gender:**   1. Male   2. Female

17. **Age **(years) ______________

18. **Family status:**   1. Single   2. Married   3. Divorced   4. Widow

19. **Country of birth**

1. Israel   2. Former Soviet Union   3. West Europe   4. East Europe

5. USA/Canada   6. South America   7. Asia/Africa   8. Other_______________

20. **Country of medical studies**

1. Israel   2. Former Soviet Union   3. West Europe   4. East Europe

5. USA/Canada   6. South America   7. Asia/Africa   8. Other_______________

21. **Primary place of work**

1. Urban clinic   2. Rural clinic/kibbutz   3. Independent clinic   4. Other ________

22. **Seniority as primary care physician (yrs)**____________

23. **Present work status**

1. Clinic director   2. Specialist in family medicine   3. Resident in family medicine   4. Other___________
